# Recent Progress and Perspectives on Functional Materials and Technologies for Renewable Hydrogen Production

**DOI:** 10.1021/acsomega.4c10407

**Published:** 2025-01-21

**Authors:** Mona Lisa M. Oliveira, Camila M. A. C. Alves, Carla F. Andrade, Diana C. S. de Azevedo, Fernanda L. Lobo, Araceli Fuerte, Paloma Ferreira-Aparicio, Concepción Caravaca, Rita X. Valenzuela

**Affiliations:** † State University of Ceará (UECE), 60714-903 Fortaleza, Ceará Brazil; ‡ Federal University of Ceará (UFC), 60355-636 Fortaleza, Ceará Brazil; § Centro de Investigaciones Energéticas, Medioambientales y Tecnológicas (CIEMAT), 28040 Madrid, Spain

## Abstract

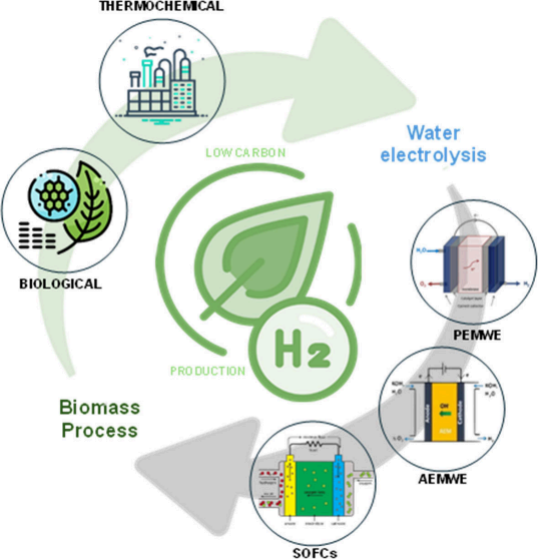

Scientists worldwide have been inspecting hydrogen production routes and showing the importance of developing new functional materials in this domain. Numerous research articles have been published in the past few years, which require records and analysis for a comprehensive bibliometric and bibliographic review of low-carbon hydrogen production. Hence, a data set of 297 publications was selected after filtering journal papers published since 2010. The search keywords in the Scopus Database were “*green hydrogen*” and “*low carbon hydrogen production and materials*”. The data were analyzed using the R Bibliometrix package. This analysis made it possible to determine the total annual publication rate and to segregate it by country, author, journal, and research institution. With a general upward trend in the total number of publications, China was identified as the leading country in research on the subject, followed by Germany and Korea. Keyword analysis and the chronological evolution of several important publications related to the topic showed the focus was on water splitting for low-carbon H_2_ production. Finally, this review provides future directions for technologies and functional materials for low-carbon hydrogen production.

## 1 Introduction

The worldwide energy landscape remains heavily reliant on fossil fuels, and not surprisingly, global CO_2_ emissions reached the highest level in history in 2023.^1^ This trend poses a major global threat as climate change intensifies. As a result, there is a large-scale sprint toward sustainability and decarbonization to mitigate the impacts of climate change by adopting sustainable and renewable energy sources.^2^ In this context, hydrogen has emerged as a promising energy carrier with the potential to play a crucial role in the energy transition.

Hydrogen stands out for its abundance, lightness, easy electrochemical conversion, and high mass-energy density.^3^ These features enable hydrogen to serve as a versatile energy carrier, which may be transported as liquid fuel via cargo ships or through pipelines. Therefore, this gas can revolutionize several sectors, including transport, energy generation and industrial processes.^4^ When used as a fuel, hydrogen forms a single byproduct (water), thus significantly reducing greenhouse gas (GHG) emissions.^5^ In this way, using hydrogen as fuel effectively contributes to the United Nations (UN) Sustainable Development Goals (SDGs), mainly SDG 7 (Affordable and Clean Energy), SDG 9 (Industry, Innovation, and Infrastructure), SDG 13 (Climate Action), and SDG 17 (Partnerships for the Sustainable Development Goals).

Although hydrogen combustion itself does not generate CO_2_, there is a global concern about the value chain required to produce this fuel. Hydrogen is classified into different “colors”, based on the renewability of this production method.^6^ Gray hydrogen, for example, is produced from fossil fuels, employing the Steam Reforming of Methane (SMR), which releases CO_2_ into the atmosphere during production. Black or brown hydrogen is produced from coal and contributes to atmospheric pollution. Blue hydrogen is also produced from SMR but embeds the capture and storage of the generated carbon. Despite being less polluting than other options, it remains insufficient for complete decarbonization.^7^

On the other hand, green hydrogen and low-carbon hydrogen are fuels that aim to produce zero- and low-carbon emissions, respectively, along their whole value chain. This is only possible if renewable energy sources, such as solar panels and wind turbines, are used for power hydrogen production, handling, and transportation.^7^ Low-carbon hydrogen production can be classified into biomass-related and water-splitting processes.^8,9^ Biomass methodologies integrate biological procedures, such as biological water–gas shift (BWGS) reaction, dark fermentation (DF), and photofermentation (PF), while thermochemical processes encompass gasification, pyrolysis, and liquefaction. The water-splitting category includes techniques such as electrolysis, thermolysis and photolysis.^2^

Several studies in Brazil,^10^ Spain,^11^ Africa,^12^ the Philippines,^13^ and South Korea^14^ have already assessed the prospects for using low-carbon H_2_ as a key element in decarbonization. Like all emerging energy sources, there are challenges related to transportation, production cost, infrastructure development, and the skilled labor force for large-scale hydrogen production. Nonetheless, projections already show that this renewable energy vector will overcome the challenges of the energy transition in the long term.^10^ This is confirmed by the high demand and considerable increase in studies aimed at new technologies for its production, storage, and use, mainly water electrolysis studies and the development of new functional materials such as advanced and effective electrolyzers.^15^

Due to numerous studies on the optimization field in renewable hydrogen production, bibliometric analysis becomes an easy option to verify trends for each purpose. Through this analysis, it is possible to track the growth trend of articles and journals in the area under study as well as the centers with the largest volume of publications in the area, which documents have received most citations, in addition to other parameters. Several published studies have already used bibliometric techniques on the topics of hydrogen storage,^16^ production,^17^ sustainability and challenges,^18^ and security,^19^ among others. A recent bibliometric study based on water electrolysis for hydrogen production was also conducted.^20^ However, the research did not focus on functional materials used for hydrogen production and was based on studies published until 2023. In addition, numerous studies have already been published in this present year, and these recent publications also need to be cataloged and analyzed in a new comprehensive review of hydrogen low-carbon production.

Among the bibliometric analyses published in the H_2_ production area, an article by Shiva Kumar and Himabindu (2019)^21^ stands out, which has already been cited more than 1,300 times to date and was published in the journal *Materials Science for Energy Technologies*. In this publication, the authors discuss the recent advancements in Proton Exchange Membrane (PEM) water electrolysis and further improvements in PEM water electrolyzer development for commercially viable hydrogen production purposes. Another recent work, by Shiva Kumar and Lim (2022),^22^ has also gained significant attention, with over 200 citations. The review by Nechache and Hody (2021),^23^ which already has more than 120 citations, summarizes the latest progress in research and development of alternative and innovative materials for electrolysis cells. Finally, the work by Lokesh and Srivastava (2022),^24^ published in *Energy & Fuels*, provides a state-of-the-art description of the various strategies to be adopted for the effective electrolysis of groundwater. In this way, it is understood that bibliometric analysis is essential to realize advances in the area. However, this analysis must be combined with a critical bibliographic review and not just the use of software without the supervision and additional treatment of the researcher.

In this context, the present review article has two main parts. The first one provides a bibliometric analysis of articles in the SCOPUS database whose objectives are (i) to analyze temporal distribution patterns of journal articles related to the topic of green hydrogen, low-carbon hydrogen, materials, and productions; (ii) to showcase contributions from authors, leading countries and the most prolific research institutions; and (iii) to highlight the outcomes of the most cited articles. In addition to the bibliometric analysis, a comprehensive assessment of the research articles addressing the technologies for low-carbon H_2_ production that appeared most in the data set obtained from the SCOPUS database, i.e., the H_2_ production from biomass and water-splitting technologies, is presented. This review is especially useful for young scientists and newcomers to the field. This will help them understand research trends and discover new opportunities for the development of innovative materials for renewable hydrogen production.

## 2 Bibliometric Analysis

**Figure 1:**
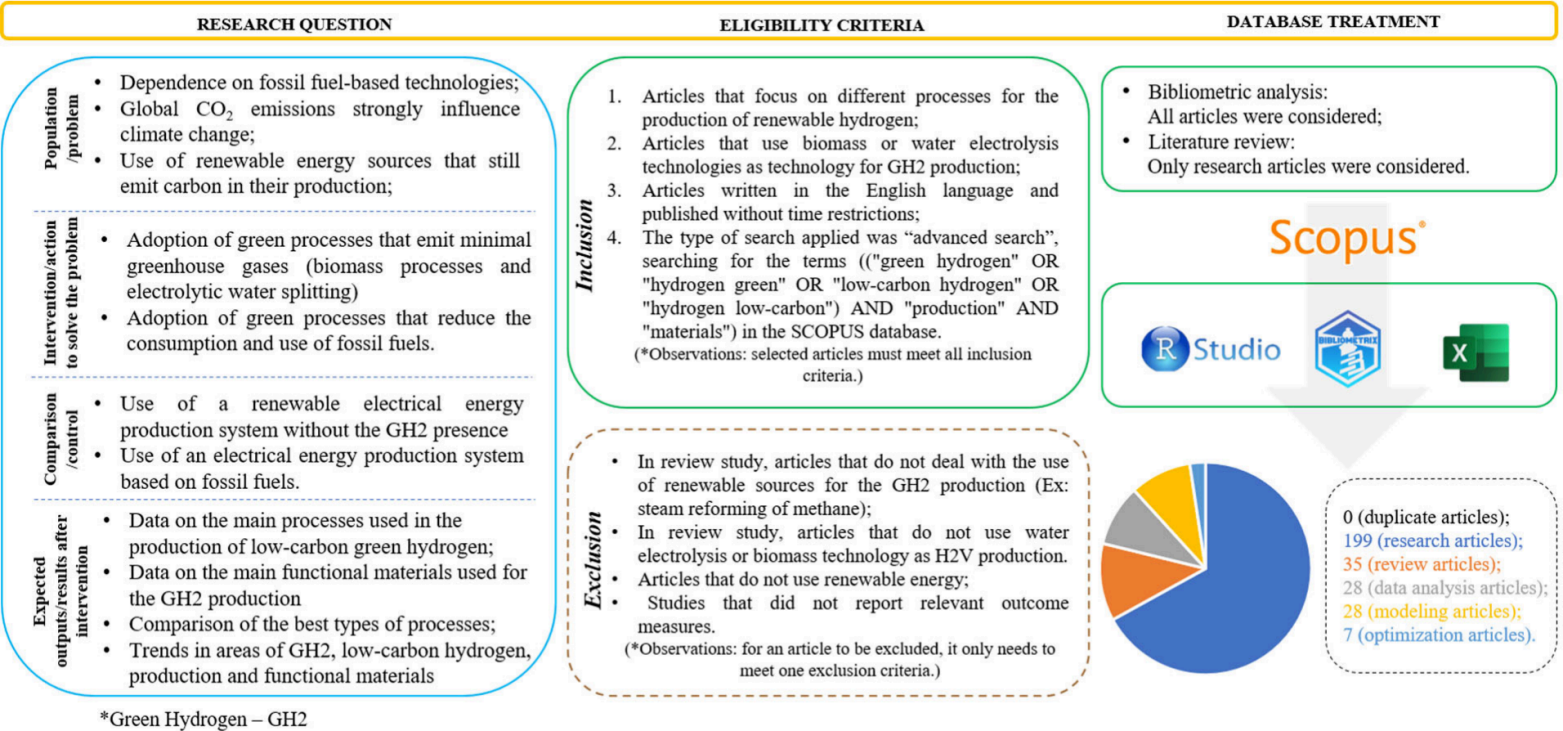
Research question and eligibility criteria adopted in the review.

To contextualize the topic under investigation, first, a bibliometric analysis was conducted to assess publications within the field. The works of Donthu et al. (2021)^25^ and Aria and Cuccurullo (2017)^26^ were used to support the bibliometric methodology, observing the different techniques and guidelines to carry out a reliable analysis (see Figure 1).

The search was conducted continuously over the specified period without time restrictions on publication using the Scopus database. The aim was to identify manuscripts published in indexed journals that would provide insights into the current state of scientific research on the proposed topic. The search was conducted during the fourth week of April 20, 2024, to capture a snapshot of publication numbers, recognizing that ongoing research activities may influence these figures over time. The refined search resulted in 297 documents, all of which were considered for bibliometric analysis (the first part of this review). For the bibliographic analysis (second part of this review), only experimental research articles were considered to verify the methods that were most used in the production of renewable hydrogen.

Bibliometric analyses are a significant aspect of research assessment, especially in the scientific and applied sectors.^27,28^ The results of the bibliometric study on green hydrogen, low-carbon hydrogen, products, and materials are presented in the following sections, indicating the most prominent research areas, keywords, affiliations, journals, and countries. Every aspect of the results was discussed, aiming to disclose research progress, trends, updates, and critical points related to the topic.

### 2.1 Publication Trend

**Figure 2:**
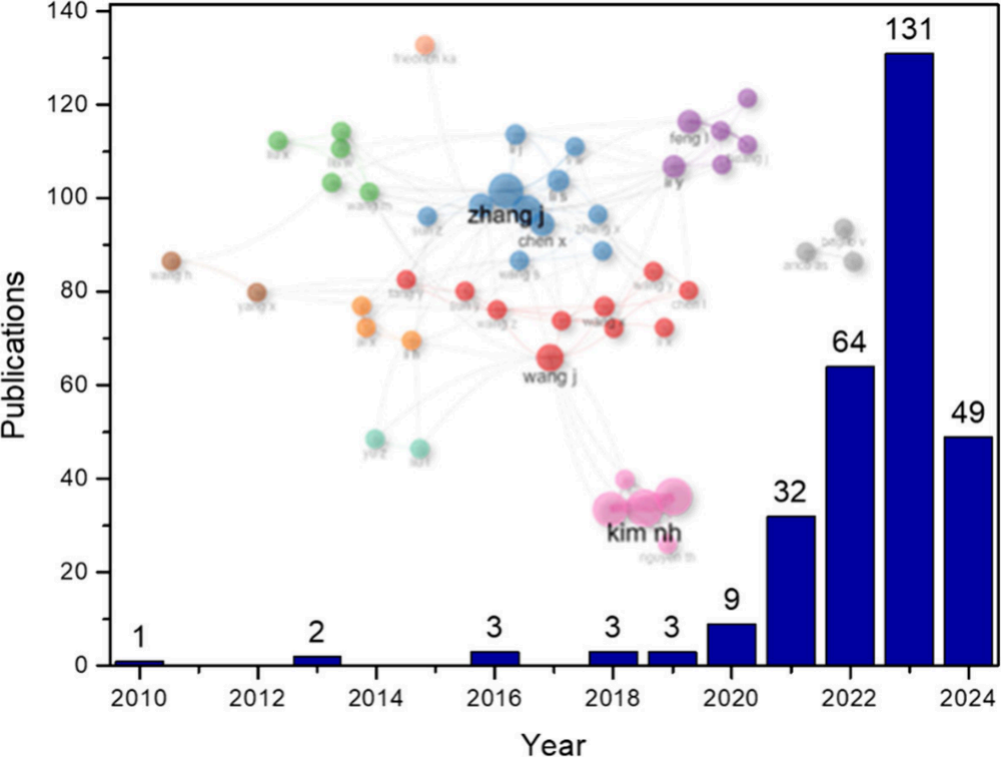
Number of articles over the years and authors collaboration.

Figure 2 shows the research trend and the evolution of publications between 2010 and 2024 with the cluster of collaborations between authors. The search encompassed the 297 documents that were considered relevant to the proposed topic.

In 2010, a single article was published, which analyzed a combined system proposed for methane steam reforming, comprising conventional hydrogen production and heat recovery waste from steel production.^29^ The other articles that appear in this data set were published in 2013, as a work by Serrano et al. (2013),^30^ whose title is “Advances in the design of ordered mesoporous materials for low-carbon catalytic hydrogen production”, and the work by Winkler-Goldstein and Rastetter (2013),^31^ entitled “Power to gas: The final breakthrough for the hydrogen economy?” Subsequently, three documents were published in 2016,^32−34^ and three more articles were published two years later (2018). The work by Li and Tsang (2018)^35^ was published in *Catalysis Science and Technology* and has already received 105 citations, the document by Tahir et al. (2018)^36^ was published in *International Journal of Energy Research*, with 32 citations, and finally, the article by Zhang et al. (2018)^37^ was published in *Energy Conversion and Management*, with 41 citations.

In 2020, the number of articles in this area began to increase, with 9 documents being published, such as the work by Verlinden (2020),^38^ published in *Journal of Renewable and Sustainable Energy* with 81 citations, and the work by Zhang et al. (2020),^39^ published in *Nano-Micro Letters* and already has 92 citations. Overall, the number of articles produced each year began to increase sharply in 2021, peaking in 2023, as can be seen in Figure 1. As the number of articles rises, the number of scientists with research interests in the topic also increases.

In 2024, the number of published articles had already surpassed the sum of papers published in 2020 and 2021, when the rise of publications in the area began, with 49 documents published by the fourth week of April 2024. Among the most recent articles, we can mention the work by Li et al. (2024),^40^ which has already received 11 citations and addresses the exploration and design of industrial water separation catalysts for large-scale green hydrogen production. The work by Wei et al. (2024),^41^ states that low-carbon hydrogen will be essential to achieve climate neutrality goals by 2050 and assesses the future environmental impacts of the life cycle of global H_2_ production, considering regional developments in the supply of raw materials and the electrical energy decarbonization. Thissen et al. (2024)^42^ explore alkaline water electrolysis in their paper, highlighting its ongoing potential for large-scale green hydrogen production. Additionally, they delve into a new material investigation that could increase efficiency.

### 2.2 Most Relevant Sources and Authors

Table 1 shows the 15 documents of the set with the highest number of citations, also showing the journal’s *h* factor, the number of articles per journal, the year in which it was first published considering this data set, the CiteScore for the year 2024, the most cited article in the magazine, and the number of citations of that article.

**Table 1:** 15 Sources Have the Highest Number of Citations^a^

	Journal	TC	TP (%)	CiteScore 2024	H_Index	PY	The most cited article (DOI reference)	Times Cited	Publisher
**1**	*Chemical Engineering Journal*	168	11 (3.70%)	21.5	6	2021	https://doi.org/10.1016/j.cej.2021.130048	56	Elsevier
**2**	*Journal of Materials*Chemistry A	138	13 (4.38%)	22	6	2013	https://doi.org/10.1039/D1TA09932A	38	Royal Society of Chemistry
**3**	*Applied Catalysis B: Environmental*	159	5 (1.68%)	37.9	5	2021	https://doi.org/10.1016/j.apcatb.2022.121312	68	Elsevier
**4**	*Energies*	109	12 (4.04%)	5.5	5	2021	https://doi.org/10.3390/en14133772	56	MDPI
**5**	*Nanoscale*	65	6 (2.02%)	13.6	5	2019	https://doi.org/10.1039/C9NR00663J	24	Royal Society of Chemistry
**6**	*ACS Applied Energy Materials*	123	7 (2.36%)	9.5	4	2019	https://doi.org/10.1021/acsaem.9b01392	78	American Chemical Society
**7**	*ACS Applied Materials and Interfaces*	73	8 (2.36%)	15.7	4	2021	https://doi.org/10.1021/acsami.2c08246	27	American Chemical Society
**8**	*Advanced Energy Materials*	95	4 (1.35%)	42.6	4	2022	https://doi.org/10.1002/aenm.202301920	30	Wiley-Blackwell
**9**	*Applied Energy*	108	6 (2.02%)	21.1	4	2021	https://doi.org/10.1016/j.apenergy.2020.116270	57	Elsevier
**10**	*Catalysis Today*	65	5 (1.68%)	11.9	4	2022	https://doi.org/10.1016/j.cattod.2021.09.015	33	Elsevier
**11**	*Electrochimica Acta*	25	6 (2.02%)	12.8	4	2021	https://doi.org/10.1016/j.electacta.2022.141582	8	Elsevier
**12**	*International Journal of Energy Research*	110	5 (1.68%)	7.2	4	2016	https://doi.org/10.1002/er.6487	54	Hindawi
**13**	*Journal of Power Sources*	68	5 (1.68%)	15.9	4	2020	https://doi.org/10.1016/j.jpowsour.2019.227563	23	Elsevier
**14**	*ACS Applied Nano Materials*	34	5 (1.68%)	7.9	3	2023	https://doi.org/10.1021/acsanm.2c04580	21	American Chemical Society
**15**	*Advanced Materials*	69	3 (1.01%)	45,5	3	2023	https://doi.org/10.1002/adma.202305074	49	Wiley-Blackwell

a Note: TC = Total Citations; TP = Total Publications; PY = Publication Year and MDPI = Multidisciplinary digital publishing institute.

The journal with the largest number of citations was the *Chemical Engineering Journal*, reaching 168 citations, it is also one of the journals with the highest *h* index, but it is not the largest number of publications in the field, falling behind the *Journal of Materials Chemistry A*, which also has the same *h* index. Among the journals featuring the latest publications, *Energy & Fuels* stands out. It debuted in this document set in 2021, has published 3 items, and has received 14 citations.

The top three authors, according to Bibliometrix and Scopus, who appear with the greatest number of publications in this set of documents are Nam Hoon Kim, Joong Hee Lee, and Duy Thanh Tran. All of them are affiliated to the Jeonbuk National University (Republic of Korea), with *h*-indexes above 40. Their research output in this data set has received over 230 citations. The most cited items describe catalysts intended for hydrogen evolution reactions (HER) and oxygen evolution reactions (OER) in an alkaline environment.

### 2.3 Most Relevant Affiliations and Number of Articles by Country

Regarding the number of documents by country, these data can be seen in Table 2, which presents the number of articles by country, the number of citations, and the percentage of articles with cooperation between other countries.

**Table 2:** Number of Documents Per Country, Total Number of Citations Per Country, Percentage of Publications with Cooperation, and the Most Productive Institution in the Country Corresponding^a^

Country	TC	TP	SCP	%SCP	The most prolific research institution
**China**	820	74	53	71.62	Chinese Academy of Sciences
**Germany**	359	24	17	70.83	Deutsches Zentrum für Luft- and Raumfahrt (DLR)
**Korea**	354	27	2	7.41	Jeonbuk National University
**Australia**	231	6	2	33.33	University of New South Wales
**United Kingdom**	225	15	8	53.33	Imperial College London
**Singapore**	151	2	0	0.00	National University of Singapore
**Switzerland**	136	1	1	100.00	Paul Scherrer Institut
**Spain**	127	11	4	36.36	Consejo Superior de Investigaciones Científicas
**India**	113	23	19	82.61	Academy of Scientific and Innovative Research (AcSIR)
**Italy**	85	16	6	37.50	Istituto di Tecnologie Avanzate per l’Energia
**Pakistan**	73	5	1	20.00	Lahore University of Management Sciences
**USA**	71	13	10	76.92	Texas A&M University
**France**	52	7	4	57.14	CNRS Centre National de la Recherche Scientifique
**Czech Republic**	49	4	2	50.00	University of Chemistry and Technology, Prague
**Canada**	33	4	1	25.00	University of Alberta
**Japan**	28	4	3	75.00	Shaanxi University of Science and Technology

a Note: TC = Total Citations; TP = Total Publications and SCP = Single Country Publications.

As can be seen, China dominates the ranking with 74 documents, also leading in the number of citations, followed by Germany in citations and Korea in publications. Switzerland has published just one article but has already garnered 136 citations. This publication, featured in *Energy and Environmental Science*, quantifies both present and future costs alongside the environmental impacts of hydrogen production systems.

Table 2 highlights countries with robust international collaboration as well as those with less international engagement, such as Korea and Singapore. It is worth highlighting Spain as a potential country in investigations into the production of renewable Hydrogen, with 127 citations in total, where the *Consejo Superior de Investigaciones Científicas* is the institution that most stands out. The international collaboration advantages are not limited to the expansion of the network, the exchange of knowledge, and the sharing of skills but also to a strategy for scientific dissemination and knowledge mobility.

### 2.4 Most Cited Documents

This bibliometric research presents the most referenced works in the low-carbon hydrogen production field. Table 3 shows the 15 most cited documents.

**Table 3:** Most Cited Documents Consider the Set of Data Analyzed^a^

Ranking	Authors	Title	TC	Journal	Year	ref
**1**	Song Lin Zhang (Zhang et al., 2021)	Engineering Platinum–Cobalt Nanoalloys in Porous Nitrogen-Doped Carbon Nanotubes for Highly Efficient Electrocatalytic Hydrogen Evolution	151	*Angewandte Chemie International Edition*	2021	(^44^)
**2**	Tom Terlouw (Terlouw et al., 2022)	Large-scale hydrogen production via water electrolysis: a techno-economic and environmental assessment	136	*Energy and Environmental Science*	2022	(^43^)
**3**	Molly Meng-Jung Li (Li and Tsang, 2018)	Bimetallic catalysts for green methanol production via CO_2_ and renewable hydrogen: a mini-review and prospects	105	*Catalysis Science and Technology*	2018	(^35^)
**4**	Shucong Zhang (Zhang et al., 2020)	2D Co-OOH sheet-encapsulated Ni_2_P into tubular arrays realizing 1000 mA/cm^2^-level-current-density hydrogen evolution over 100 H in Neutral water.	92	*Nano-Micro Letters*	2020	(^39^)
**5**	P. J. Verlinden (Verlinden, 2020)	Future challenges for photovoltaic manufacturing at the terawatt level	81	*Journal of Renewable and Sustainable Energy*	2020	(^38^)
**6**	Li Wang (Wang et al., 2019)	High-performance anion exchange membrane electrolysis using plasma-sprayed, nonprecious-metal electrodes	78	*ACS Applied Energy Materials*	2019	(^45^)
**7**	Wei Liu (Liu et al., 2022)	Tuning the atomic configuration of the CO–N–C electrocatalyst enables highly selective H_2_O_2_ production in acidic media	68	*Applied Catalysis B: Environmental*	2022	(^46^)
**8**	Fabian Scheepers (Scheepers et al., 2021)	Temperature optimization for improving polymer electrolyte membrane-water electrolysis system efficiency	57	*Applied Energy*	2021	(^47^)
**9**	Graham Palmer (Palmer et al., 2021)	Life-cycle greenhouse gas emissions and net energy assessment of large-scale hydrogen production via electrolysis and solar PV	57	*Energy and Environmental Science*	2021	(^48^)
**10**	Wang Dongliang (Dongliang et al., 2021)	Green hydrogen coupling with CO_2_ utilization of coal-to-methanol for high methanol productivity and low CO_2_ emission	56	*Energy*	2021	(^49^)
**11**	Deger Saygin (Saygin and Gielen, 2021)	Zero-emission pathway for the global chemical and petrochemical sector	56	*Energies*	2021	(^50^)
**12**	Kyoung Ryeol Park (Park et al., 2021)	Copper-incorporated heterostructures of amorphous NiSe_*x*_/Crystalline NiSe_2_ as an efficient electrocatalyst for overall water splitting	56	*Chemical Engineering Journal*	2021	(^51^)
**13**	Steffen Kiemel (Kiemel et al., 2021)	Critical materials for water electrolysis at the example of the energy transition in Germany	54	*International Journal of Energy Research*	2021	(^52^)
**14**	Taotao Gao (Gao et al., 2023)	Understanding the atomic and defective interface effect on ruthenium clusters for the hydrogen evolution reaction	53	*ACS Catalysis*	2023	(^53^)
**15**	Daqin Guan (Guan et al., 2023)	Identifying a universal activity descriptor and a unifying mechanism concept on perovskite oxides for green hydrogen production	49	*Advanced Materials*	2023	(^54^)

a Note: TC = Total Citations.

Among the most cited papers, the type of H_2_ production technology most sought after is electrocatalytic water splitting by means of different processes. One of the most cited articles was a review article, also showing the importance of this type of research in academia.

### 2.5 From Bibliometric to Bibliographic Analysis

The bibliometric analysis of articles from the Scopus database offers an overview of the most frequently published studies within the specified keywords and filters. However, the information presented in this type of survey cannot be measured based only on the number of citations, most relevant research institutions in the area, authors who have published the most, and others. In many cases, good research or publication should only be assessed by the research itself, regardless of whether it is going to be cited or not cited. Due to this, the researcher must curate beyond the raw data provided by the software, thus performing a bibliographic analysis beyond bibliometric analysis.

Thus, research articles must be selected and analyzed from the data set for this more comprehensive review. In this study, of the 297 articles found, 7 deal with optimizations in systems aimed at green hydrogen produced, and 28 works include case studies or economic analyses of H_2_ on a large-scale use. Another 28 works use modeling, through either DFT calculation or life cycle analysis (LCA) to verify materials or better ways to produce low-carbon hydrogen. The other 35 articles are review articles that address the topic of study. As expected, most articles are research articles (199) where it is important to highlight the large number of works that research new materials using different technologies for renewable H_2_ production. However, despite using the filter for “only articles that address low-carbon hydrogen production”, some articles were still found that address the steam reforming of methane, for example. This and other works will not be considered for this literature review.

Among the selected articles, it was observed that the most studied production technology in the data set was the electrolytic splitting of water, whose literature review will give greater focus to the next topics. In addition, other processes were also studied among the 199 research articles, which will be discussed in more depth in the following section.

## 3 Technologies for H_2_ Production from Biomass

One of the main ways to produce hydrogen sustainably is through the numerous technologies that use biomass, such as thermochemical, biological, and electrochemical methods. Thermochemical conversion is the most established method for the production of hydrogen from biomass. The process was established based on a similar process performed on nonrenewable biofuels, such as steam methane reforming (SMR), adapted to biomethane (renewable) use. The three main thermochemical routes are gasification, pyrolysis, and aqueous phase reforming (APR). Gasification is a highly endothermic process conducted in an oxygen-deficient environment at ∼1000 °C, utilizing an oxidizing agent to produce synthesis gas, which also contains hydrogen. The process is categorized based on the oxidizing agent used, including gasification with air, oxygen, or steam. Steam gasification is regarded as the most effective method for H_2_ production from biomass, as it yields a high percentage of H_2_ in the gas (40%), a higher H_2_/CO ratio, and fewer impurities compared to air gasification. Additionally, steam reforming (SR) serves as a complementary purification step, enhancing the synthesis gas composition during steam gasification and further increasing H_2_ yield.^55^

Another thermochemical route for biomass conversion is pyrolysis, which operates similarly to gasification but at lower temperatures (400 to 800 °C), under pressures up to 5 bar, and without requiring an oxidizing agent. Pyrolysis is classified based on operating temperature and reaction conditions: slow/conventional pyrolysis (450 °C), produces high biochar yields fast pyrolysis (450–600 °C) with high heating rates (∼300 °C/min) and short residence times, generates up to 75% bio-oil by weight; and ultrafast/flash pyrolysis (above 600 °C) with extremely high heating rates (>1000 °C/s) and very short residence times (<1 s), which maximizes gas production. A third pathway for hydrogen production is aqueous-phase reforming, where oxygenated compounds are converted into hydrogen. In this process, feedstock molecules dissolve in water and react with water molecules at relatively low temperatures (<270 °C) and high pressures (up to 50 bar). APR is particularly well-suited for oxygenated hydrocarbons derived from biomass with a 1:1 C/O ratio and water solubility, such as methanol, ethanol, ethylene glycol, glycerol, glucose, and similar compounds.^56^

Compared with thermochemical processes, biological conversion occurs at lower temperatures, between 30 and 60 °C and pressures of 1 atm, reducing energy costs. Biological methods can be divided into the biological water–gas shift (BWGS) reaction, dark fermentation (DF), and photofermentation (PF). The BWGS reaction uses photoheterotrophic bacteria using carbon monoxide as a carbon source. These microorganisms can produce H_2_ (along with CO_2_) in the dark, oxidizing CO and reducing H_2_O through an enzymatic pathway. Dark fermentation uses anaerobic organisms (such as microalgae or specific bacteria) that are kept in the dark at temperatures between 25 and 80 °C, or even at hyperthermophilic temperatures (>80 °C), depending on the strains. Finally, photofermentation is the most recent biological process used for H_2_ production. PF is catalyzed by nitrogenases in purple nonsulfur bacteria to convert organic acids or biomass into hydrogen from solar energy in a nitrogen-deficient medium. It is worth noting that all biological methods also require optimizing numerous parameters and the development of technologies to be financially competitive and have the potential for practical application and commercialization.^57^

Finally, electrochemical methods are also included in the production of renewable hydrogen, and among them are the proton exchange membrane electrolysis cell (PEMEC) and the microbial electrolysis cell (MEC). PEMECs and MECs are commonly used for biobased molecules such as ethanol and glycerol. The conversion of organic matter occurs at the anode by an oxidation reaction, releasing protons. A reduction reaction occurs at the cathode, allowing the formation of H_2_. In MEC systems, the oxidation of organic matter to produce H^+^ is accomplished using electrochemically active microorganisms as catalysts.^58^

The research articles described above discuss different technologies for renewable hydrogen production, especially for H_2_ production from biomass sources. Table 4 summarizes the technologies and materials used for this purpose.

**Table 4:** Technologies for H_2_ Production from Biomass Sources

Process	Materials	Results	Authors	ref
**Dark fermentation**	Black liquor is used as substrate and mud as inoculum. Immobilization of anaerobes on graphene (GN), hydroxyapatite (HN), and graphene/hydroxyapatite nanoparticles (GHN)	H_2_ yield of 0.579 mol/mol glucose	Tawfik et al. 2021	(^59^)
**Dark fermentation**	Milk processing wastewater (MPWW) was used as substrate (S) and waste sludge was used as inoculum (I). Various substrate pretreatments: ultrasonic, thermal, chemical, and enzymatic hydrolysis	Better conditions resulted in 254 mL of H_2_ production	Bouchareb et al. 2024	(^60^)
**Dark fermentation and bacteria photofermentation**	Bacteria such as clostridia, enterobacter, rhodobacter and bosea	Accumulated hydrogen volume of 169 mL, and hydrogen yield of 0.63 mol H_2_/mol glucose	Wang et al. 2022	(^61^)
**Fermentation with alternation of light and darkness**	Microalgae of the *Chlorella vulgaris* sp. type as biomass	H_2_ production in the order of 52 mL	Ardo et al. 2024	(^62^)
**Co-fermentation**	Beer grains and cheese whey (CW) as biomass. Use of CO_2_ produced in fermentation for better biosynthesis of microbial metabolites	Bio-H_2_ recovery was maximum with 30 g/L cod (181.35 mL/day)	Sarkar et al. 2021	(^63^)
**Microwave-assisted pyrolysis from straws**	Wheat straw, rice straw, and corn stover as biomass. Catalysts based on carbon materials and NiO/C composites	Total gas yield of 58.7% and a H_2_ yield of 25.4% in the best condition	Yue et al. 2024	(^64^)
**Biomass pyrolysis, catalytic reforming of pyrovapores, and biochar heat treatment processes**	The tandem catalyst of biochar + spinel NiAlO + biocarbon reforming	The result of 91% by weight of synthesis gas with a proportion of H_2_ + CO greater than 83 vol %	Yang et al. 2024	(^65^)
**Biomass pyrolysis and catalytic steam reforming of bio-oil**	A mixture of agricultural crop residues as raw material–LaNi_0.5_CO_0.5_O_3_ perovskite as a catalyst	Stable bio-oil conversion (80%) and average H_2_ yield (60%)	Singh et al. 2024.	(^66^)
**Biomass gasification via synergistic deoxygenation and decarburization**	Deoxidizing and decarburizing of CaO-Fe (Ca:Fe > 1.0) materials	H_2_ yield of 68.16 mol/S/kg of biomass and H_2_ concentration of up to 93.58 vol %	Sun et al. 2023	(^67^)
**Bio-oil steam reforming via fixed-bed tubular reactor**	LaNi_0.5_M_0.5_O_3_ perovskite catalysts (M = Co, Cu, and Fe)	Maximum H_2_ yield (79%) and bio-oil conversion (94%)	Singh et al. 2023	(^68^)
**Bioethanol steam reforming**	YNi/Mo_2_TiC_2_T_*x*_ (yNi/MTC) materials as a catalyst	The best condition achieved a H_2_ utilization efficiency of up to 95.6% and almost complete ethanol conversion	Shi et al. 2023	(^69^)
**Ethanol electro-reforming**	Adenine-derived noble carbons as catalytic supports and PtRu/ANC as electrocatalysts	H_2_ was produced with 100% faradaic efficiency (in cathode)	Rodríguez-Gómez et al. 2023	(^70^)
**Plastic visible light-driven reforming**	Mesoporous ZnIn_2_S_4_ photocatalyst has been applied in photoreforming of polylactic acid (PLA), polyethylene terephthalate (PET), and polyurethane (PU)	The H_2_ yield was 142.8 μmol g/h. No CO_2_ was detected during the photocatalytic process	Zheng et al. 2023	(^71^)
**Microbial electrolysis cells (MECS)**	Flowable cathode in MECS with nickel-loaded activated carbon (Ni/AC) powders	The MEC with a Ni_4_/AC_0.125_ flow cathode produced comparable H_2_ production rates and 40% higher than the blank	Moreno-Jimenez et al. 2023	(^72^)

As highlighted, one of the most studied technologies are those related in data set to the use of biomass to produce hydrogen, whether through dark fermentation, with the use of biomass^63^ or microalgae,^62^ or by means of pyrolysis^64^ and gasification.^67^ It is noteworthy that bio-oil^68^ and ethanol^69^ steam reforming have been investigated as ways of generating hydrogen with low carbon emissions, making production greener. Finally, a study using microbial electrolysis cells is shown in Table 4. A possible emerging area may be related to these microbial electrolysis cells, as analyzed in the work of de Moreno-Jimenez et al. (2023).^72^

## 4 Water Electrolysis for Green H_2_ Production

Electrocatalytic water splitting is one of the most studied technologies for hydrogen production. Its fundamentals have been known since 1789, and by the beginning of the 20th century, more than 400 industrial alkaline water electrolyzers were in operation worldwide. In the 1990s, a renewed interest in water electrolysis was stimulated by hydrogen, which was regarded as a green energy carrier for renewable energy sources like wind and solar power. However, it has been only in the past decade that a significant increase in global interest in water electrolysis has appeared, with the adoption of ambitious national climate protection programs. Water electrolysis is considered a key issue for sector coupling and is expected to make an important contribution to reducing greenhouse gas (GHG) emissions close to net zero by 2050. Although the expression “green hydrogen” is widespread in all current publications related to hydrogen production from water, some years ago was not commonly used, as shown in the bibliometric analysis covering recent entries since 2010.

Alkaline water electrolysis is a well-established mature technology for industrial hydrogen production up to the multimegawatt range. However, it is less efficient than other newer approaches. This section will review the most advanced technologies: proton exchange membrane water electrolysis (PEMWE), anion exchange membrane water electrolysis (AEMWE), and solid oxide electrolysis (SOWE), focusing on materials development.

### 4.1 Proton Exchange Membrane Technology

Proton exchange membrane water electrolysis (PEMWE) has emerged as an up-and-coming technology for sustainable H_2_ production. It is a favorable and reliable option for efficient hydrogen splitting that provides interesting advantages such as the system design’s compactness, high current density, exceptional efficiency achieved, and rapid system responses with minimized gas crossover rates. However, their widespread application relies heavily on developing high-performance and cost-effective HER and OER electrocatalysts.

Electrocatalysts containing Pt, Ir, and, in some cases, Ru have been demonstrated to be able to reduce overpotentials for HER and OER, respectively. This excellent behavior contrasts with their large cost and limited availability, which remains the main shortcoming of widespread commercial application. Considerable research effort has been devoted to developing non-noble metal catalysts for PEM applications, although their substantially lower efficiency makes the good performance of noble metals outweigh their cost.^73,74^ Simple transition metal oxides have garnered significant attention as a result of their claimed performance in acidic conditions.^75^ The most recent strategies for materials research are based on first-principles calculations such as Density Functional Theory (DFT),^76^ machine learning, and optimization approaches to reduce the overpotential for metal-based and metal-free OER and HER electrocatalysts.^77,78^

**Figure 3:**
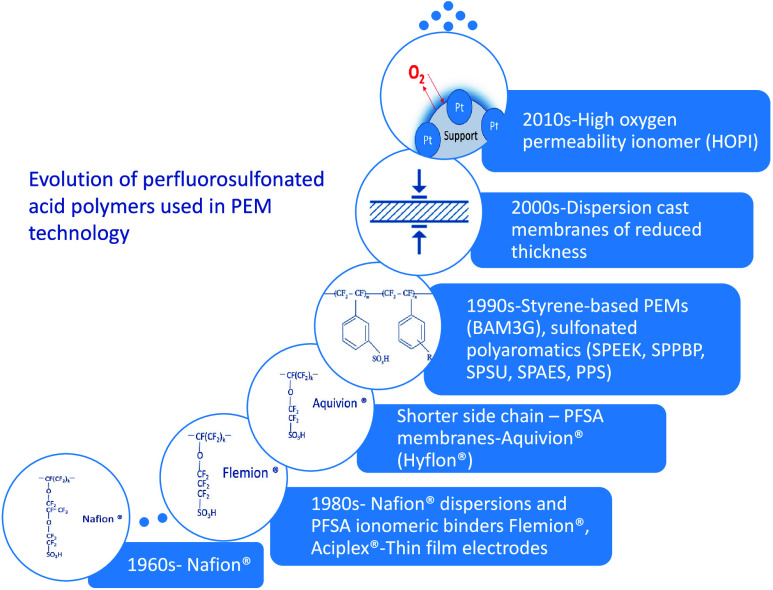
Some membranes used in PEM.^80−86^

On the other hand, the electrolyte used in these systems is also a main concern. The initial idea of using an organic cation exchange membrane as a solid electrolyte in electrochemical cells was first described in 1959 by Grubb, a scientist working for General Electric Company.^79^ The resins used for this purpose were changed in the following years, but it was in 1962 that the perfluorosulfonic acid membrane, Nafion, led to a breakthrough in this technology. The most used polymers in proton exchange membrane electrolysis cells over the years are compiled in Figure 3.

The use of fluoropolymers for PEM is currently key for the hydrogen electrochemical technologies, and no alternative is foreseen to be able to substitute them in the short term. The primary motivation for using hydrocarbon polymers in PEM cells is the need to reduce the widespread use of perfluorinated compounds. In this regard, the application of graphene oxide-based films as proton exchange membranes has been recently proposed as a viable option.^87−89^

Although PEM water electrolysis technology is becoming a mature technology, its main limitation lies in its cost and high operating voltage use. Among the different component costs, bipolar plate materials and manufacturing account for 40%–60% of a water electrolysis stack.^90−93^ Materials used for bipolar plate (BP) manufacturing must have excellent strength, low resistivity, high thermal conductivity, and low hydrogen permeability. In particular, the anode-side plates are expected to operate under a demanding corrosive environment (typically, 1.6–2 V, pH 2–4, 50–90 °C, and O_2_-saturation).^94^ Their corrosion has a large impact on the whole PEMWE stack since dissolved metal ions would migrate to the membrane/electrodes, and both the ionic conducting and catalytic performance would be affected, resulting in reduced I–V performances and increased cell voltages at a given electrolysis current.^95^ As a reference for the most recent targets established for these components, they should have a lifetime no shorter than the stack target lifetime, e.g., 80,000 h, corrosion current density, electrical resistivity, and interfacial contact resistance should be as low as possible, even lower than in PEM fuel cells (∼1 μA cm^–2^) considering the long service lifespan required for electrolyzers in H_2_ production without high overpotentials and ohmic losses.

Titanium is a state-of-the-art candidate for PEMWE plates. Its good anticorrosion properties in the acidic medium due to the formation of a protective oxide film on its surface act as a barrier to the external corrosive environment. In addition, its low density (4.5 g cm^–3^) allows for achieving high gravimetric power density of the stacks, however, there are some concerns regarding its use.^90−95^ Other metals, such as stainless steel, aluminum, copper and nickel alloys have been investigated using suitable coatings.^94−98^ Therefore, materials that avoid overpotentials and ohmic losses of electrolyte, membrane, and electrode resistances have been studied. In addition, the effect of temperature must be considered, since PEM at low temperatures can slow down the reaction kinetics.^99^

### 4.2 Anion Exchange Membrane Technology

**Figure 4:**
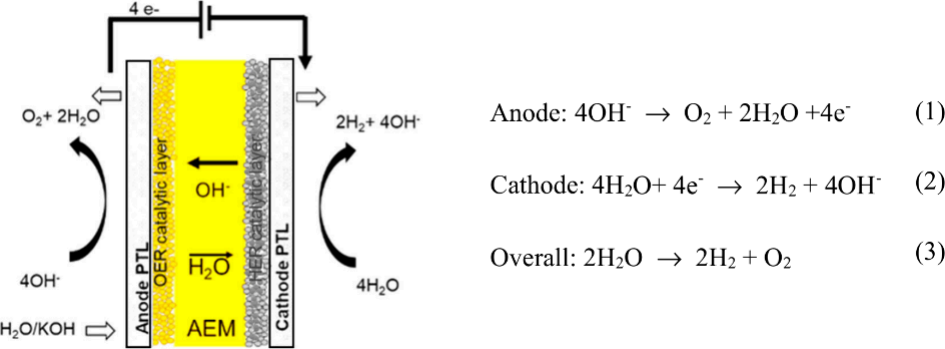
General schematic diagram of the AEMWE.

Anion exchange membrane electrolysis (AEMWE) is an emerging technology that combines the benefits of two technologies: alkaline water electrolysis (AWE) and proton exchange membrane water electrolysis (PEMWE). It replaces the nonconductive porous diaphragm of the AWE for a nonporous anion conductor solid membrane, enhancing safety and eliminating the need for highly concentrated KOH solutions. As a result, it operates more efficiently at high pH levels without critical raw materials (CRM) such as platinum group metals (PGM). Additionally, it can operate at higher current densities thanks to lower ohmic resistance, allowing for the use of smaller devices similar to those of PEM devices.^100,101^ AEMWE involves two electrochemical half reactions occurring in the electrolyte: the HER at the cathode, where the protons and electrons recombine, and the OER at the anode. These reactions are represented in eqs 1–3 together with the scheme of the AEMWE shown in Figure 4.

The current challenge in developing AEM catalysts lies in optimizing their activity, chemical composition, and stability when integrated into the AEM system. For instance, while water splitting theoretically requires a voltage of 1.23 V, actual operating voltages typically range from 1.7 to 2.3 V. This discrepancy is primarily due to unfavorable properties of the electrode materials, such as overpotentials and inadequate ion and gas dispersion, as well as system-related factors like liquid electrolyte concentration and other resistances.^101−106^

Regarding the materials used in the process, platinum group metals (PGMs), particularly platinum, exhibit the highest exchange current density. However, their high cost and limited availability make them impractical for large-scale electrolyzer production. PGM-free electrocatalysts, while more economical, generally have lower mass-specific activity compared to PGMs. This necessitates a higher loading on the membrane electrode assembly (MEA), which in turn leads to significant ohmic resistance losses.^107^

The 3*d*-transition metals (Ni, Co, Fe, Mo, Mn, and W) used have been considered for many years as catalysts for water splitting. The interest of these metals and alloys is based on their partially full d-orbitals which allow for easy participation in reagent-mediated electron transfer, they present different oxidation states and ability to form complexes, which may decrease overpotentials and increase energy efficiency.^108,109^

#### 4.2.1 HER and OER Catalysts

Hydrogen Evolution Reaction (HER) on different metals is one of the most widely investigated reactions. Even though there are currently good-performing electrodes, there is great interest in finding electrodes that further reduce the overpotential for HER to minimize energy consumption during water electrolysis. In alkaline media, the HER mechanism occurs in three reaction steps (Volmer step, Heyrovsky step, or Tafel step).^101,104,106^

The rate-limiting step of the whole mechanism is the formation of the initial hydrogen intermediate by H_2_O dissociation and subsequent H_ad_ adsorption on the catalyst surface. In AEM electrolysis HER requires more energy to break the covalent O–H bonds of water, being a crucial step that determines the HER activity.^103^

Regarding the OER reaction, the anodic reaction (oxygen evolution) of the overall water-splitting reaction in alkaline media has an equilibrium potential at standard conditions of 1.23 V versus reversible hydrogen electrode (RHE).
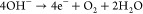
4

For this reaction to take place at reasonable rates is necessary to apply higher overpotentials than those needed for the HER. This is due to the sluggish kinetics of the OER due to its reaction mechanism that involves 4 proton-coupled electron transfer steps.^101,110,111^

An enormous number of materials have been tested to determine their electrocatalytic activity toward the OER in half-cells using a three-electrode setup. Following, some of the materials are investigated for use as anionic catalysts for AEM electrolyzers. A review of HER and OER materials can be seen in Table 5.

**Table 5:** Materials Used Most in the HER or the OER Reactions

Reaction	Materials	Properties	ref
**HER**	Ni and Ni-based alloy with metals or oxides (Fe, Cu, Ti, Mo, Co, Sn, MoO_2_, CeO_2_)	Highest corrosion resistance in alkali media (hydrogen binding energy is close to that of Pt)	(106−108, 112−114)
**HER**	Transition metal (TM) nitrides – NiN/Ni	Enhanced HER activity by doping with Mo or Co. Metallic matrix presents low overpotentials, however, their stability decreases with time.	(115)
**HER**	NiMo, NiW, CoMo and CoW alloys	Superior activity of NiMo and CoMo in alkaline fuel cells.	(116, 117)
**HER**	Nonmetallic elements (C, N, S, O, P and B)	Alter the adsorption-free energy of reaction intermediates, leading to fast water dissociation	(118)
**HER**	Carbonates (Mo_2_C/NC@0.5Ni) and S- and Se-based compounds	Pt-like properties and HER activity due to the shift of the d-band center	(119−122)
**HER**	Spinel oxides (AB_2_O_4_), perovskite oxides (ABO_3_), Rudddlesden-Popper type oxides (A_*n*+1_B_*n*_O_3*n*+1_)	Low cost, earth abundance, easy synthesis, composition and structural diversity, and flexible tenability	(123−125)
**OER**	Electrodeposited Ni and Fe alloys	Aging and activations reduce the OER overpotential by thickening the Fe-doped Ni-oxo-hydroxide layer	(126, 127)
**OER**	Ni, Co and Fe oxides	Oxidation of Co and Fe to higher oxidation states also increases the OER activity	(128, 129)
**OER**	LaCoO_3_	Surface reconstruction by synthesizing an amorphous layer on the surface (LSCF-0) by Co reduction to Co^2+^ enhances the performance	(130, 131)
**OER**	LaNiO_3_	The created lanthanum deficiency facilitates the segregation of NiO from the initial matrix forming an interface between the perovskite and NiO phase, resulting in a 4.5-fold increase in OER activity	(132)
**OER**	Co-based electrode supported on FeO_*x*_H_*y*_ (Fe@Co)	The overpotential was low and showed the lowest average degradation rate.	(133, 134)

As mentioned, studies seek to replace precious metals in the HER and the OER catalysts. One of the most investigated materials used as an HER catalyst is Ni, which has the highest corrosion resistance in alkali media, and its hydrogen binding energy is close (but lower) to that of Pt. The cooperative interaction of metals with different hydrogen binding energy (HBE) could emulate the activity of PGM electrocatalyst.^108,110^ Various authors have studied Ni-based alloys with metals or oxides (Fe, Cu, Ti, Mo, Co, Sn, MoO_2_, CeO_2_) to prevent Ni hydride formation and increase their stability.^107^ Alloys containing Mo seem to have high activity and present high corrosion resistance in alkaline media and good electrical conductivity and thermal stability. This high catalytic activity of NiMo alloys is attributed to the synergistic effect of adjacent resulting in unsaturated *d*-orbitals similar to Pt. Incorporation of nonmetallic elements such as C, N, S, O, P, and B can also alter the adsorption-free energy of reaction intermediates helping the fast water dissociation, although most works reported better HER performance in acidic than in alkaline media.^118^ In the case of (Co_4_N@NC), N-doped C suppresses the surface oxidation of CoN_*x*_ in alkaline media and enhances the electrical conductivity.^115^

Similar to nitrides, carbides exhibit Pt-like properties and HER activity due to the shift of d-band center.^119^ It is claimed that Mo_2_C nanocrystalline coupling with Ni has been successfully encapsulated into a Ni-doped carbonaceous network (Mo_2_C/NC@0.5Ni) and Mo_2_C/NCNT@0.5Ni as inexpensive transition metal catalysts for AEM electrolyzers. However, when it was tested both as bifunctional catalysts, this structure provided an overall structural flexibility and ion/electron transport kinetics. TM oxide-based materials are promising candidates for catalyzing the HER due to their composition and structural diversity, which offers electronic and crystal structure flexibility with various chemical and physical properties. The low cost, earth abundance, easy synthesis, composition and structural diversity, and flexible tenability make them attractive for HER catalysis since efficient and cost-effective catalysts are critical to widespread the hydrogen as a clean energy carried. Based on their structural features, metal oxides are classified into single oxides, spinel oxides (AB_2_O_4_), perovskite oxides (ABO_3_), Rudddlesden-Popper type oxides (A_n+1_B_n_O_3n+1_), metal hydroxides (oxo), specially structured metal oxides, oxide-containing hybrids. Detailed information about the HER behavior of some of these oxides was summarized by Zhu et al.^123^ Despite these above-mentioned HER electrocatalysts having been tested, there are still no electrocatalysts that offer kinetics superior to or similar to that of platinum.^124,125^

Regarding OER, many materials have been tested to determine their electrocatalytic activity. Electrodeposited Ni and Fe alloys with different compositions and crystallographic orientations were also studied. Their chemical composition strongly influences their initial electrochemical performance. Aging and activations reduce the OER overpotential by thickening the Fe-doped Ni-oxo-hydroxide layer. The layer Ni^3+^/Ni^2+^ capacity and the ratio Fe/Ni determine the apparent OER kinetics. The alloy must develop a thick Ni-rich active surface layer (large number of Ni active sites) and efficient sites (large Fe/Ni ratio: 0.2 < *x* < 0.4).^127^ The electrocatalytic activity of Ni, Co, and Fe oxides toward the OER based on the overpotential follows the order: Ni > Co > Fe, inversely to the bond strength OH-M_2+δ_ (0 ≤ δ ≤ 1.5) order. Ni oxidation (Ni^2+^ to Ni^3+^) produces Ni(OH)_2_ transformation in NiOOH. Oxidation of Co and Fe to higher oxidation states also increases the OER activity.^128^

#### 4.2.2 Other AEMWE Components

**Figure 5:**
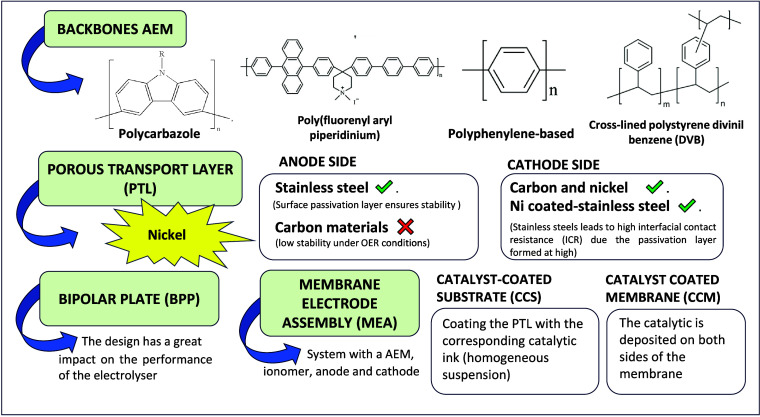
Other essential components in the AEMWE electrolysis.

The AEM allows the migration of hydroxide anions from the cathode to the anode and physically separates both compartments to avoid the crossover of reagents and products, hence it must fulfill several requirements: (i) ionically conductive; (ii) thermal, mechanically, chemically and electrochemically stable; (iii) low cost, easy to process and produced by sustainable processes.^101^ AEM consists of polymer backbones where the anion exchange cation functional groups, which confer the anion selectivity, are anchored.^101,102,135−139^Figure 5 shows this type of component and the others that can be observed in AEMWE electrolysis.

The porous transport layer (PTL) is a major contributor to the performance of AEMWE.^140−143^ PTLs in AEMWE as compared to those of PEMWE have the advantages of reduced cost and a dramatic reduction impact on CRMs. The preferred material for the PTL on both the anode and cathode side is nickel. Figure 5 also presents a summary of the most commonly used materials in the anode and cathode.^144−148^

About the membrane electrode assembly (MEA), this system contains the AEM, ionomer, anode, and cathode. There are two common methods to deposit the catalytic layer: producing catalyst-coated substrates (CCS) or catalyst-coated membranes (CCM). The CCSs are prepared by coating the PTL with the corresponding catalytic ink, homogeneous suspension of the catalyst, and binder. In contrast, catalytic layers are deposited on both sides of the membrane in CCMs. Mechanical or hot pressing is used when the MEA is placed between the PTL. While a hot press is usually used for PEM cells, this option is not good for AEM since mechanical force and high-temperature damage the membrane.^149^ In summary, it is essential to carry out several studies to optimize the MEA design since, as previously mentioned, this architecture directly influences the final water-splitting results. An efficient design minimizes ohmic resistances, while poorly designed electrodes can create barriers that increase resistance and reduce efficiency. The cell assembly must ensure uniform water distribution to the catalytic electrode and the rapid removal of gaseous products. Dispersion problems can result in the formation of gas bubbles that block active areas, increasing the overpotential.

Concerning HER and OER catalysts, the choice of specific materials for the cathode and anode as well as the uniformity in the application of the catalyst on each electrode (in addition to the adequate assembly of the components) directly impacts the current density. A well-designed electrode ensures that all reactive areas effectively participate in the reaction, avoiding local efficiency losses. Studies to focus on new catalytic materials and homogeneous deposition techniques are fundamental since slow kinetics may require higher overpotentials to achieve the desired reaction rate. The oxygen evolution reaction is particularly challenging due to its slow kinetics as it involves a multiple electron transfer process. This significantly contributes to voltage losses. Although HER is faster compared to OER, nonprecious catalysts exhibit higher overpotentials than platinum, especially in alkaline systems.

### 4.3 Solid Oxide Water Electrolysis Technology

Among the different water electrolysis technologies, the high-temperature steam electrolysis process, via Solid Oxide Electrolysis Cells (SOECs), is the most efficient electrolysis method and it has attracted much attention over the past decade. This technology, which operates at high temperatures (700–800 °C), shows a very high efficiency (>95%) and low energy consumption to split water into hydrogen (<40 kWh/kg H_2_) due to advantageous thermodynamics and enhanced HER and OER kinetics.^150,151^ It means a strong reduction in hydrogen cost, as power consumption is the main contributor to the cost of hydrogen in the electrolysis processes.

Although high temperature is beneficial in terms of efficiency and performance, it causes structural degradation and makes long-term applications difficult. In this sense, an intensive search for new materials and cell configurations is taking place to decrease the operating temperature and mitigate cell degradation processes. Nevertheless, long-term tests of SOEC stacks have been performed for above 4000 h, with 70% steam conversion and high performances, with a current density of 0.85 A/cm^2^ and about the thermoneutral voltage (1.3 V).^152−154^ They lead to a low degradation of <2% after 1000 h of continuous operation.

**Figure 6:**
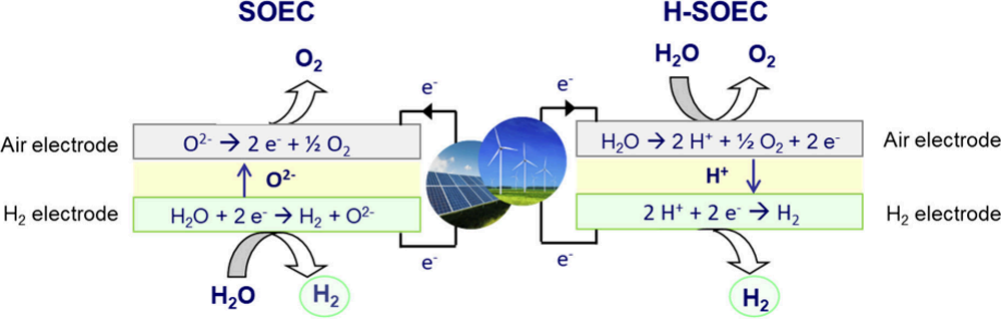
Different types of solid oxide electrolysis for green hydrogen production.

A solid oxide electrolysis cell is constituted of two electrodes: an anode or air electrode and a cathode or hydrogen electrode, both separated by a dense ionic conducting electrolyte. Depending on the nature of the ion conducted through the electrolyte, two categories of Solid Oxide Electrolysis are distinguished, the traditional oxide ion-conducting electrolysis cells (SOECs) and proton-conducting electrolysis cells (H-SOECs) that have attracted increasing interest due to their lower operating temperatures (450–700 °C).^155^ As shown in Figure 6, in the case of a solid oxide electrolysis cell (SOEC), water is supplied at the cathode side (H_2_ electrode) and it is reduced into H_2_ and oxide-ion (O^2–^) that is conducted through the electrolyte to the anode side (air electrode) to form O_2_ by oxidation. On the contrary, in the case of proton-conducting solid oxide cells (H-SOEC) water is supplied at the anode side (air electrode) and forms protons, using renewable electricity (for green hydrogen).

Protons are conducted through the electrolyte to the cathode side (hydrogen electrode) and produce hydrogen. Due to the better proton transport facilitated by the electrolyte, compared with the oxide-ion conducting counterparts, in which ionic transport is realized by less mobile oxygen anions, the H-SOECs are able to operate effectively at lower temperature ranges.^156^ Another advantage of these electrolyzers over oxygen-conducting cells (SOECs) is the formation of pure hydrogen at the fuel electrode, not having to require its separation from steam.

HER and OER reactions take place at the triple phase boundaries (TPBs) where the ionic phase (oxygen ion or proton conductive), the electrical phase (e-conductive), and the gas (hydrogen release or steam supply) coexist. For that, the electrolyte must be highly conductive for ions (O^2–^ or H^+^ for SOEC or H-SOEC, respectively), electrically insulating to prevent electronic conduction, and sufficiently dense to avoid gas transport between electrodes. On the other hand, HER and OER electrodes should be porous materials with mixed ionic–electronic conductivity and catalytically active to allow good performance.^157^ Finally, interconnect materials also play a key role in the stacking of electrolyzers; they connect individual cells to each other, providing mechanical support and conductivity for the stack. They work as a current collector, completing the electrical circuit of the system, and act as a physical barrier between the hydrogen electrode and the air electrode of adjacent cells. They should show high electrical and thermal conductivity and high stability under both oxidizing and reducing atmospheres. All of the materials that form the SOEC electrolyzer must show suitable thermal and chemical compatibility over time to avoid cracks and cell degradation to ensure sufficient durability. The main degradation processes observed in these systems are the delamination at the interface electrode/electrolyte, which can be avoided by optimizing the microstructure and electrodes, gas diffusion through the fuel electrode due to the high humidity, and degradation of electrodes due to microstructure coarsening during sintering, migration of elements, or formation of secondary phases.

Several materials have been researched for the different solid oxide electrolyzer components; Table 6 briefly summarizes the latest progress in the development of new materials for traditional oxide ion-conducting electrolysis cells (SOECs).

**Table 6:** Materials Most Used as SOEC Components

Components	Materials	Properties	ref
**Electrolyte**	8YSZ	High ionic conductivity and stability at high temperature. Limited conductivity at lower temperatures	(158−162)
**Electrolyte**	Doped ScSZ	Higher stability to low temperatures	(163, 164)
**Electrolyte**	Doped ceria	Higher stability to low and medium temperatures	(165−167)
**Electrolyte**	LSGM and other perovskite-type doped lanthanum gallates	Higher conductivity to low and medium temperatures but suffers from Ga evaporation under reducing conditions or the formation of insulating layers working with Ni	(168−170)
**HER catalysts**	Ni-YSZ	Electrode degradation under electrolysis conditions, Ni agglomeration and depletion	(171)
**HER catalysts**	BaCO_3_-infiltrated Ni-YSZ fuel electrode	Increased the current densities and reduced the interfacial polarization resistance between electrolyte-electrode, improved the cell performance under steam electrolysis operation	(172)
**HER catalysts**	@-GDC (Ni, Cu, Co, Au and Mo)	Higher ionic conductivity of gadolinium doped ceria (GDC), its mixed conduction properties and compatibility with YSZ electrolyte (avoiding the use of intermedia layers and reducing cell manufacturing costs)	(173−176)
**HER catalysts**	NI-YSZ/YSZ/LSM, NI-YSZ/YSZ/GDC/LSCF-GDC, NI-YSZ/YSZ/LSCF-GDC, or AU-MO-NI-GDC/YSZ/GDC/LSCF	The performance of Ni-cermets fuel electrodes in SOEC operation modes has been tested in several studies using different configurations with good results.	(177−182)
**HER catalysts**	Perovskite-Based Materials, doped Lanthanum Strontium Manganites (LSM) and La–Sr Ferrites (LSF).	Mixed ionic-electronic conductivity with a remarkable stability under oxidizing and reducing atmospheres at high temperature	(183−190)
**HER catalysts**	Lanthanum-substituted strontium titanates (LST perovskites) with cerium oxide impregnation and nickel or doping with different cations	Ca and Fe impregnation, for example, improves the conductivity and the catalytic activity	(191−193)
**HER catalysts**	Transition metal-exsoluted catalysts.	Increases the concentration of oxygen vacancies and promote its contact with steam during the electrolysis process, improving the electrocatalytic activity of the electrodes and consequently the final cell performance	(194−199)
**OER catalysts**	Cobalt and strontium doped perovskites and Co-based and Sr-doped lanthanum manganites	Higher electrocatalytic activity, chemical stability, conductivity and good cost-effectiveness. Operational conditions, such as high current densities for long time, are the main cause of degradation of the air electrode.	(200, 201)
**OER catalysts**	Composite LSM-YSZ	Enhance the electrochemically active area, extended triple-phase boundary (TPB) and enhanced electrolysis cell stability	(202−204)
**OER catalysts**	Mixed ionic and electronic conducting (MIEC)—as a LSCF material	High electrical and ionic conductivity and oxygen diffusion properties that achieves higher electrocatalytic activity than LSM reference material	(205−207)
**OER catalysts**	Mo-doped SrFeO_3-δ_, LaBaMn_2_O_5+δ_ or PrBaMn_2_O_5+δ_ double perovskite	High ionic conductivity in air, suitable electronic conductivity, excellent catalytic activity and low cost. Adding Co or Cu improves its catalytic activity	(208−214)
**OER catalysts**	Ruddlesden–Popper lanthanide nickelates (Ln_2_NiO_4+δ_, Ln = La, Nd, Pr).	Lower polarization resistance and higher current density, higher electrochemical performance and stability than LSCF and moderate thermal compatibility with common electrolytes materials. Ni is frequently substituted with Co, and La with Sr and Ca	(215−217)
**Interconnect**	Chromium alloys: Crofer 22 APU or 22H, Ducralloy, CrFe5, CFY, AISI430, etc.	Conductivity loss by oxidation and the cathode poisoning by chromium evaporation under water electrolysis conditions	(218, 219)
**Interconnect**	Protective coatings with perovskites or Mn–Co spinels	High cost-effectiveness and performance.	(220)

Over the past decades, many efforts have been made to decrease the operating temperature to the low or intermediate range to prevent cell degradation. In this sense, proton-conducting solid oxide cells (H-SOECs) provide an excellent basis for advances in high-temperature solid oxide devices. The facilitated ionic transport in proton-conducting electrolytes, mainly BaZrO_3_-based electrolytes, enables these cells to operate at significantly lower temperatures (below 500 °C), offering high efficiency and excellent performance. Novel functional materials and technological strategies to optimize the H-SOECs have been recently summarized.^221,222^

## 5 Challenges and Perspectives

### 5.1 Opportunities for Biomass for Low-Carbon Hydrogen Production

A viable alternative to the natural gas steam reforming technique is the use of biogas, or biomethane, which has the extra benefit of being renewable feedstock economically generated that lessens pollution by eliminating the emission of methane into the atmosphere. Landfill gas recovery facilities and anaerobic digesters for biowaste treatment, including municipal solid waste (MSW), fertilizers, and energy crops, are great opportunities for biogas and biomethane production. In addition to reducing landfill waste, biogas production also yields nutrient-rich fertilizer as a byproduct. In biogas steam reforming processes, numerous supported catalysts have been tested. The materials that are mostly used as catalysts in reforming are nickel-based, such as Ni/Al_2_O_3_, due to their low cost and satisfactory efficiency. The main problem with Ni-based catalysts is that they are subject to several types of deactivations, including sintering, oxidation, carbon deposition, and sulfur poisoning. Intensive research efforts are currently being made to improve the performance and lifetime of alumina-supported nickel catalysts.^223^

Ruthenium, nickel, and iron nanoparticles have demonstrated good catalytic activity in steam reforming. Entrapping nanoparticles for hydrogen production rather than chemically produced ones might save costs. Nanoparticle recycling has been successfully shown in several investigations. Although it has been suggested that employing green nanoparticles might potentially lower operational costs, it is evident from the data collected from several studies that adsorption is still expensive even when using green nanoparticles that have been manufactured.^224^ The inexpensive and ecologically friendly nanoparticle development should improve the perspectives of hydrogen production via biogas steam reforming.

### 5.2 Opportunities for Microbial Electrolysis Cell (MEC)

Another viable technology for low-hydrogen production is the Microbial Electrolysis Cell (MEC), which carries a unique benefit because it demonstrates good potential to convert biomass and waste organics into high-quality H_2_ while concurrently solving environmental challenges, such as wastewater treatment. MECs employ electroactive bacteria in the anodic chamber and reduce the external voltage needed for H_2_ evolution. Compared to abiotic water splitting that requires ∼1.8 V-2.0 V to overcome the thermodynamic barrier, MEC leverages the chemical energy in organic compounds. As a result, much less external voltage (∼0.6–1.0 V) is required, and even such a small voltage need can be met when a traditional cathode is replaced with a photocathode or by deploying an *in situ* power management circuit.^225,226^

Regarding the materials used in the anode, some properties should be considered such as high conductivity, high corrosion resistance, the possibility of bacterial attachment, and large surface area. In addition, the material should be economical and sustainable. Carbonaceous materials, such as graphite rods, carbon brushes, graphite felt, plain carbon cloth, and activated carbon, are the most preferred for making anode electrodes. Regarding the cathode, materials such as titanium, silver mesh, and nickel foam are investigated. Some carbonaceous materials are also considered as cathode materials but they are found to have slow HER rate due to their high potential and platinum is a potential material that can be used to minimize this problem.^227^

A comparison was made by Tang et al. (2022)^226^ of H_2_ production recovery rates (HPRs) among all reported MEC cathodes by catalyst. HPR is an important indicator of MEC performance, indicating the volume of H_2_ produced per MEC reactor volume over time. The highest HPR of 4.2 m^3^/m^3^ day using new types of catalysts (stainless steel, copper, manganese, and molybdenum) was lower than the absolute maximum rates that Pt and Ni foam achieved in the previous decade (between 17.8 and 50 m^3^/m^3^ day). While novel catalysts have been demonstrated to exhibit suitable performances, it can be difficult to directly compare these catalysts when data are collected from separate experiments under different conditions. Therefore, studies conducted with uniformly designed experiments are necessary to ensure that results are comparable.

In summary, MEC is a promising technology, as it can convert organic waste into hydrogen and other value-added chemicals with only a small energy input. However, optimization of sustainable hydrogen production should be carried out more carefully because, in addition to the materials that can be varied (e.g., anode, cathode, membrane), other parameters can affect MEC performance, including bacteria, substrate composition, and the designs and configuration of the MEC.

### 5.3 Opportunities for Water Electrolysis

Concerning the splitting process used for H_2_ production, water electrolysis (whether by PEM, AEM, or SOEC technology) is an energy-intensive process that benefits from the use of catalysts. Because the canonical HER catalyst for PEM electrolysis is Pt and the OER catalysts are IrO_2_ and RuO_2_, an important research focus for low carbon hydrogen production has been the development of catalysts that rival scarce metals in performance but with reduced or eliminated metal loading. As seen in this article, recent materials research landscape in this area can be visualized in many ways. Photocatalysts and nanomaterials (nanoparticles, nanosheets, nanocomposites) are a new trend in materials for hydrogen production.^228,229^

The most optimal HER electrocatalysts can reduce energy and cost compelled for electrochemical water splitting through the decreased overpotential. Therefore, research development to produce efficient nonprecious electrocatalysts for HER is critically important and challenging. The electrocatalysts in HER are Ni and Co-based, including nickel-based alloys, nickel-based phosphides, cobalt oxides, cobalt phosphides, cobalt sulfides, cobalt selenides, and other transitional metal-supported nanomaterials such as molybdenum disulfide supported carbon nanotubes (MoS_2_/CNTs), nickel phosphide supported CNTs (Ni_2_P/CNTs), cobalt doped iron disulfide CNTs (FeS_2_/ CNTs), tungsten dioxide supported carbon nanowires (WO_2_/C), Co–Fe nanoalloys, and nickel-yttria-stabilized zirconia (Ni-YSZ).^230,231^

AEMWE can play a key role in the predicted enormous growth of green hydrogen technology with essential R&D advances in the coming years. The reality, however, is that AEM membranes have chemical and mechanical stability problems, leading to unstable lifetime profiles. Moreover, performance is not yet as good as expected, mostly due to low AEM conductivity, poor electrode architectures, and slow catalyst kinetics.^232^ Another challenge to be overcome by AEMWE is the low stability of most membranes in alkaline media and the need to increase their ionic conductivity to operate effectively with a reduction in the alkalinity of the feed solution to ultimately use water and even seawater.^233^

Some other key design parameters essential for commercialization are (i) stable alkaline OER catalyst design with high electronic conductivity and minimal surface reconstruction during operation. These catalyst layers must be applied to the MEA with scalable, industrial techniques; (ii) ionomer oxidation mitigation strategies should be developed, this could lead to other creative catalyst layer designs.^232,233^ Transition metal catalysts with excellent electrocatalytic properties can contribute to improving performance for AEMWE. Nevertheless, NiFe-based catalysts remain promising to be used in the anode due to their active OER. Catalysts should maintain their stability for long periods but also must operate effectively when operated at high current densities and intermittent power supply, since power fluctuation from wind and solar energy may deteriorate their performance.^233^

Concerning the PTLs or GDLs, the future challenges are related to the optimization of morphology and porosity to enhance gassing while maintaining the conductivity and mechanical structure. Another aspect that should be investigated is the reduction of their thickness to decrease costs while maintaining the mechanical stability. Another way to reduce costs is the substitution of nickel for stainless steel, although it might reduce the performance a bit, as indicated previously. Some of the proposed activities to fill the real gaps were summarized by the International Renewable Energy Agency (IRENA) in its 2020.^234^

In contrast to other water electrolysis technologies, more widely commercialized, SOECs operate at much higher temperatures, which confers higher efficiency and current densities, which are of great interest for the future demand of clean hydrogen production. However, their commercial availability is mainly limited by aspects, such as lifetime and power cost. In this sense, future research is mainly focused on the development of novel materials with high chemical stability, new coating technologies, and the optimization of operating conditions to improve the performance and address degradation phenomena.

## 6 Conclusion

A bibliometric analysis using the R Bibliometrix tool was performed, where 297 publications were selected after filtering journals published since 2010. This analysis allowed us to determine the total annual publication rate and segregate it by country, author, journal, and research institution. With an overall upward trend in the total number of publications, China was identified as the current leading country in research on the topic, followed by Germany and Korea. The analysis of several parameters related to the low-carbon hydrogen production topic showed that the focus has been on water splitting for renewable H_2_ production.

Massive progress has been made in the functional materials field and technologies for low-carbon hydrogen production in recent years, and efforts to find better and cheaper catalysts have brought this technology closer to mass production and operation. However, there are significant challenges that have not yet been fully addressed yet. This review identifies major barriers to ultimate commercial large-scale hydrogen production by water electrolysis. First, the development of non-noble metal OER electrocatalysts with high activity and long-term stability performance in acidic media remains a challenging area of research and development. For the HER, there are various efficient non-noble metal electrocatalysts available in acid media. However, for the OER, most of the efficient OER catalysts are Ir and Ru-based electrocatalysts which have higher dissolution resistance in acidic conditions. For non-noble-metal-based electrocatalysts, most of them cannot survive under such conditions. Thus, there is a clear need for the development of stable and robust non-noble metal OER electrocatalysts. Second, there is limited knowledge of detailed catalytic mechanisms, especially for transition-metal-based HER and OER electrocatalysts. The intrinsic active site of electrocatalysts cannot be completely determined based on the descriptor of turnover frequency. Recently, non-noble-metal-based carbides, phosphides, and chalcogenides have drawn great attention due to their high performance for the OER in alkaline media. However, the nanostructured electrocatalysts undergo composition and structural transformations during the reaction under the OER conditions.

Therefore, understanding the structural transformation is required to determine the real active phases and sites. Gaining insight into the detailed mechanism, structural transformation, and real active sites is critical for the rational design of optimal performance catalysts. Integration of in situ characterization techniques and theoretical modeling is an advanced approach to gain insights into the structural transformation, reaction intermediates, and catalyst reaction pathways. Third, it is difficult to directly compare various nanostructured catalyst materials based on the performance descriptors due to the different mass loadings of the catalysts on the electrode and the different materials of the substrate, which may affect the electron transfer rate by different electrochemical measurement methods. More effective electrocatalyst screening strategies are needed to establish a standard evaluation protocol for effective comparisons of the performances of catalysts from various research groups. Nevertheless, the surge of recent interest in nanostructure and lattice oxygen engineering of catalysts is expected to lead to new advances in the design of active, stable, and low-cost OER and HER electrocatalysts for the mass commercialization of water electrolysis-based hydrogen production.
